# Immediate Effects of Pre-bronchoscopy Pulmonary Physiotherapy on Post-procedure Pulmonary Function in Individuals Undergoing Bronchoscopy

**DOI:** 10.7759/cureus.110150

**Published:** 2026-06-02

**Authors:** Manish Kumar S, Kalpana AP, Santha Kumar S

**Affiliations:** 1 Cardio-Respiratory Physiotherapy, KMCH College of Physiotherapy, Coimbatore, IND; 2 Cardio-Pulmonary Physiotherapy, KMCH College of Physiotherapy, Coimbatore, IND; 3 Pulmonology, Kovai Medical Center and Hospital, Coimbatore, IND

**Keywords:** bronchoscopy, diaphragmatic breathing, oxygen saturation, pulmonary physiotherapy, respiratory rate

## Abstract

Introduction

Pulmonary function plays an essential role in procedural safety and respiratory stability. Flexible bronchoscopy is an important diagnostic and therapeutic procedure; however, procedure-related complications, including hypoxemia, oxygen desaturation, bronchospasm, and laryngeal spasm, may impair pulmonary function and post-procedure recovery. Breathing exercises may improve respiratory performance by enhancing breathing mechanics and strengthening respiratory muscle function. However, evidence regarding the role of pre-bronchoscopy pulmonary physiotherapy remains limited.

Methods

This quasi-experimental study included 30 individuals undergoing flexible bronchoscopy with bronchoalveolar lavage (BAL). Participants were divided into a control group (n = 15) and an experimental group (n = 15). The experimental group received pre-bronchoscopy pulmonary physiotherapy interventions, including relaxed diaphragmatic breathing exercises, thoracic mobility exercises, the controlled cough technique, and low-intensity mobilization, whereas the control group received routine hospital care. Outcome measures, including peak expiratory flow rate, oxygen saturation, respiratory rate, and chest expansion, were assessed before the intervention and three to four hours following bronchoscopy.

Results

Within-group analysis demonstrated significant improvement in oxygen saturation (SpO_2_) (93 ± 2.3 to 96 ± 1.9%, p = 0.001) and a significant reduction in respiratory rate (20 ± 1.4 to 18 ± 1.0 breaths/min, p = 0.001) in the experimental group. The control group demonstrated a significant improvement in peak expiratory flow rate (328 ± 61.2 to 310 ± 59.8 L/min, p = 0.001) and significant improvements in chest expansion at all three levels. Between-group analysis showed statistically significant differences in oxygen saturation (p = 0.001) and respiratory rate (p = 0.001), favoring the experimental group. Peak expiratory flow rate did not demonstrate a statistically significant difference between groups (p = 0.85). Chest expansion showed statistical significance only at the axillary level (p = 0.019).

Conclusions

In our cohort, pre-bronchoscopy pulmonary physiotherapy demonstrated short-term beneficial effects on selected pulmonary parameters, such as oxygen saturation and respiratory rate, in individuals undergoing bronchoscopy.

## Introduction

The integrity of pulmonary function is essential for procedural safety, as it is required to maintain adequate gas exchange and airway clearance. Flexible bronchoscopy, introduced in 1968, has evolved into a vital diagnostic and therapeutic tool for respiratory conditions. It is widely utilized in clinical practice as it enables direct visualization of the lower airways. It has several applications, including bronchial brushing, bronchial washing, bronchoalveolar lavage and biopsies, cryobiopsies, endobronchial biopsies, and transbronchial biopsies [1]. The common indications for diagnostic flexible bronchoscopy include haemoptysis, lung opacities of unknown cause, consolidation, cavity lesions, new pulmonary nodules, suspected bronchogenic carcinoma, and evaluation of foreign body aspiration [2].

According to the Indian Bronchoscopy Survey (2017), approximately 70,000 flexible bronchoscopy procedures were performed in the preceding year. Lignocaine jelly was the most commonly used method for nasal lignocaine administration (81.2%), while nebulized lignocaine was utilized for topical anesthesia, either routinely or occasionally, in 72.4% of cases [3]. Despite its wide clinical application and favorable safety profile, bronchoscopy may result in temporary alterations in pulmonary function, which may affect post-procedure recovery.

General complications include persistent hypoxemia, characterized by an oxygen saturation (SpO₂) below 90% during the procedure, psychomotor agitation, arrhythmias, bronchospasm, and laryngeal spasm. In a study involving 1,035 patients, complications occurring during the procedure or within 24 hours after bronchoscopy were reported, depending on the patient’s pulmonary status [4]. During the procedure, the arterial partial pressure of oxygen may decrease by approximately 10-20 mmHg, which can increase the risk of respiratory complications. This reduction in oxygen levels may occur due to hypoventilation, right-to-left shunt, impaired diffusion, and ventilation-perfusion mismatch. The severity of desaturation can be influenced by baseline lung function, existing comorbidities, sedative use, and procedure-related factors [5].

A study reported that bronchoscopy can cause a significant and acute decline in pulmonary function parameters, including forced vital capacity, forced expiratory volume in one second, and peak expiratory flow rate [6]. Airway pressure during fibreoptic bronchoscopy shows minimal pressure changes in spontaneously breathing patients. However, the procedure may temporarily reduce vital capacity and forced expiratory volume due to airway obstruction and suctioning. The transient increase in positive end-expiratory pressure and functional residual capacity may prevent hypoxemia during the procedure when patients are receiving 100% oxygen. After removal of the bronchoscope, bronchospasm and laryngospasm may contribute to post-procedural hypoxemia and desaturation [7].

Breathing exercises can improve respiratory performance by enhancing breathing mechanics and strengthening respiratory muscle function. Diaphragmatic breathing exercise has been shown to increase chest expansion, reduce respiratory rate, and improve the efficiency of oxygenation. It serves as a useful non-pharmacological intervention for individuals with stress-related disorders as well as those with chronic respiratory diseases [8,9]. The potential strategy of low-intensity exercise may be more feasible and acceptable for middle- to late-aged survivors [10]. Pre-rehabilitation has gained increasing attention, with most of the studies focusing on exercise as part of pre-operative management [11]. However, the available evidence regarding pre-pulmonary physiotherapy in individuals undergoing bronchoscopy remains limited. Most existing studies have focused on pulmonary rehabilitation in post-operative respiratory conditions and post-bronchoscopy lung volume reduction procedures rather than pre-bronchoscopy pulmonary physiotherapy [12].

Furthermore, there is limited evidence specifically investigating the immediate effects of pre-bronchoscopy pulmonary physiotherapy on post-procedure function. Techniques such as relaxed diaphragmatic breathing exercises, thoracic mobility exercises, and low-intensity mobilization can potentially minimize acute physiological alterations following bronchoscopy. However, the role of these interventions has not been investigated. This represents an important gap in the current literature. Therefore, this study aimed to investigate the immediate effects of pre-bronchoscopy pulmonary physiotherapy on post-procedure pulmonary function in individuals undergoing bronchoscopy.

## Materials and methods

Study design, setting, and timeline

This research was conducted using a pre-test/post-test, quasi-experimental design to evaluate the immediate effects of pre-bronchoscopy pulmonary physiotherapy on post-bronchoscopy pulmonary function in individuals undergoing bronchoscopy. The study was conducted at Kovai Medical Center and Hospital, Coimbatore, and enrolled individuals admitted to the bronchoscopy unit from March 2026 to April 2026.

Ethical consideration

Ethical approval was obtained from the Institutional Scientific Research Committee of KMCH College of Physiotherapy (ref no: KMCHCOPT/ISRC/02/2026).

Participants and sampling

In total, 30 individuals undergoing bronchoscopy participated in the present investigation. The sample size was estimated using G*Power software version 3.1 based on a previous study evaluating the effects of deep breathing exercise on pulmonary function parameters, using peak expiratory flow rate as an outcome measure. Using an effect size of 1.03, an alpha error of 0.05, and a power of 80%, the estimated sample size was 15 individuals per group, resulting in a total sample size of 30 individuals. Convenience sampling was used for participant recruitment by enrolling eligible individuals who met the inclusion criteria after obtaining written informed consent in Tamil and English.

Participants were informed about voluntary participation, confidentiality, and their right to withdraw from the study at any time without penalty. The selected participants were subsequently assigned alternately to the experimental group and control group, with the first eligible participant assigned to the experimental group, followed by alternate allocation of subsequent participants. Eligibility was confirmed by pulmonologists using a structured screening form (including demographics, indication for procedure, medical history, type of procedure, and exclusion criteria) administered by physiotherapists.

Eligibility criteria

Inclusion Criteria

Individuals eligible for inclusion were required to meet the following criteria: aged 18 to 70 years, of any sex, undergoing bronchoscopy with a bronchoalveolar lavage (BAL) procedure, hemodynamically stable as confirmed by pulmonologists, and willing to participate and provide informed consent.

Exclusion Criteria

**Figure 1:**
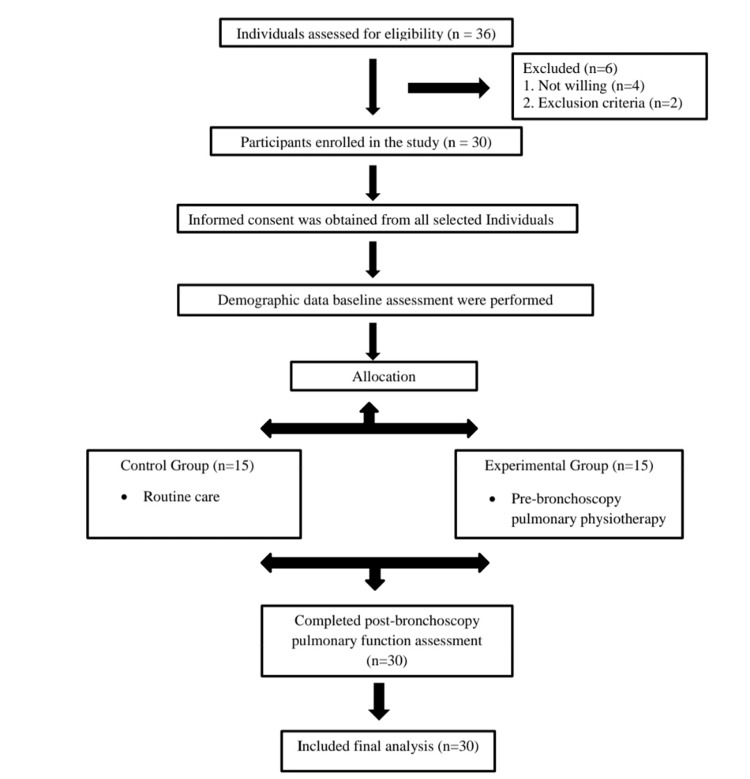
Participant flow diagram showing recruitment, allocation, assessment, and final analysis

The exclusion criteria were as follows: untreated pneumothorax, active haemoptysis, individuals with implantable cardioverter-defibrillator (ICD), severe neurological disease, acute illness, mechanical ventilation and respiratory distress, severe hypertension, and unstable cardiac conditions (including recent myocardial infarction (MI) and angina). The participant selection and allocation process is illustrated in Figure 1.

Intervention

After allocation into two groups, the experimental group received pre-bronchoscopy pulmonary physiotherapy, including relaxed diaphragmatic breathing exercises, thoracic mobility exercises, the controlled coughing technique, and low-intensity mobilization exercises aimed at improving breathing mechanics, oxygen utilization efficiency, and chest expansion. The exercises were administered three to four hours before the bronchoscopy procedure. Descriptions of each exercise are provided in Table 1.

**Table 1:** Description of exercises* ^*^[9,11]

SI. No.	Exercise	Techniques	Position	Instruction	Reps/sets
1	Breathing exercise	Relaxed diaphragmatic exercise	Semi-Fowler's position	Instructed to perform slow inspiration, enhancing abdominal expansion and minimizing upper chest movement, hold it for three seconds, followed by slow controlled exhalation	4 reps/2 sets
2	Thoracic mobility exercise	Shoulder flexion with inspiration and expiration with extension	High sitting	Instructed to raise both arms forward and upward during deep inhalation and lower the arms during slow exhalation	4 reps/2 sets
3	Thoracic mobility exercise	Thoracic flexion with expiration and extension with inspiration	High sitting	Instructed to bend the trunk forward while slowly exhaling and then extend the spine while taking a deep inspiration	4 reps/2 sets
4	Coughing technique	Controlled coughing technique	High sitting	Instructed to perform deep diaphragmatic inspiration and hold it for three seconds, followed by the controlled cough technique	-
5	Mobilization	Low-intensity mobilization exercise	Standing	Early mobilization, such as ambulation, as tolerated by the individual	4-5 minutes

The intervention session lasted approximately 30 minutes. All exercises were performed under the supervision of a physiotherapist. Appropriate safety measures were followed throughout the intervention, and vital signs were continuously monitored during the exercise session. The control group received routine bronchoscopy care as per hospital protocol. This included monitoring of vital signs such as heart rate, blood pressure, and oxygen saturation, along with any procedure-related effects. No additional pulmonary physiotherapy exercises were administered.

All participants underwent flexible bronchoscopy with BAL according to hospital protocol. Sedation was administered as per the pulmonologist's recommendations, and general anesthesia was not used in this study. The procedure duration was generally comparable among participants; however, minor variations occurred depending on individual characteristics. Potential confounding factors, including baseline pulmonary status, disease severity, and individual patient characteristics, as well as temporary post-procedure physiological changes, were considered during participant screening and interpretation of findings.

Outcomes and tools

Outcome measures were recorded before the intervention and post-intervention, four hours after bronchoscopy. Post-intervention assessment was performed four hours after bronchoscopy based on pulmonologist recommendations and participant tolerance, as immediate assessment after bronchoscopy may be influenced by transient symptoms such as nausea, the sensation of vomiting, and residual effects of sedation. All assessments were conducted by a physiotherapist.

Cough efficacy was measured using a portable peak expiratory flow meter, which reflects the efficiency of expiratory airflow. Individuals were positioned in a high sitting position and asked to exhale maximally to empty the lungs. The mouthpiece was then attached to the participant’s mouth (a disposable mouthpiece was used for each individual to minimize cross-contamination). Following this, the participant was instructed to take a deep breath and then exhale as forcefully as possible. The test was repeated for three trials, and the highest value obtained was recorded for analysis.

Oxygen saturation was measured using a pulse oximeter to assess peripheral oxygenation. It was performed with the participant in a comfortable position, and the pulse oximeter was placed on the participant’s index finger. It was allowed to stabilize for a few seconds, and the displayed SpO₂ value was recorded for analysis. Respiratory rate was assessed to evaluate the breathing pattern. The measurement was performed in a comfortable position. Initially, the pulse rate was assessed for 30 seconds by palpating the radial pulse. Without informing the participant, chest movements were observed for an additional 30 seconds to determine the respiratory rate. This method was used to ensure that the participant remained unaware, as awareness may alter the normal breathing pattern.

Chest wall expansion was measured using an inch tape to assess thoracic mobility. It was performed in a high sitting position. The inch tape was placed circumferentially around the chest at the levels of the axilla, nipple, and xiphisternal level. Participants were instructed to perform maximal inhalation followed by maximal exhalation. The difference between chest circumference at maximal inspiration and maximal expiration was recorded for analysis.

Data analysis

Statistical analysis was performed using SPSS Statistics software version 31.0.2.0 (IBM Corp., Armonk, NY). Demographic data and outcome variables were calculated using descriptive statistics. An independent t-test was used to determine the statistical significance of the difference between the groups. The paired t-test was used to compare the mean of pre-and post-intervention values within groups. A p-value <0.05 was considered statistically significant.

## Results

The mean values of age, gender, height, weight, and BMI for both groups are presented in Table 2. The intra-group analysis of the pre- and post-intervention results, outlined in Table 3, showed changes in pulmonary function in both groups. Compared to the control group, the experimental group showed a significant improvement in oxygen saturation (p = 0.001) and a significant reduction in respiratory rate (p = 0.001). Conversely, the control group's respiratory rate increased, though this change was not statistically significant (p = 0.068). A significant improvement in peak expiratory flow rate was observed in the control group (p = 0.001), whereas the reduction in the experimental group was not statistically significant (p = 0.115). Furthermore, chest expansion showed significant improvement at all three levels within the control group (p = 0.001), while the experimental group showed significant improvement at the xiphisternal level only (p = 0.017).

**Table 2:** Descriptive statistics of demographic variables in control and experimental groups SD: standard deviation

SI. No.	Variable	Values
1	Age, years, mean ± SD	57 ± 10.8
2	Gender, n (%)	Male: 18 (60%)
		Female: 12 (40%)
3	Height, cm, mean ± SD	165 ± 5.9
4	Weight, kg, mean ± SD	61 ± 5.84
5	BMI, kg/m^2^, mean ± SD	22.3 ± 2.3

**Table 3:** Comparison of pre- and post-intervention values of pulmonary parameters within the control and experimental groups SD: standard deviation; PEFR: peak expiratory flow rate; SpO₂: oxygen saturation; RR: respiratory rate; CE: chest expansion

SI. No.	Variables	Groups	Pre-test, mean ± SD	Post-test, mean ± SD	P-value
1	PEFR, L/min	Control	328 ± 61.2	310 ± 59.8	0.001
		Experimental	330 ± 55.6	315 ± 70.1	0.115
2	SpO₂, %	Control	94 ± 2.7	90 ± 3.0	0.001
		Experimental	93 ± 2.3	96 ± 1.9	0.001
3	RR, breaths/min	Control	19 ± 1.6	20 ± 2.0	0.068
		Experimental	20 ± 1.4	18 ± 1.0	0.001
4	CE axillary level, cm	Control	3.0 ± 0.9	2.0 ± 1.4	0.001
		Experimental	3.4 ± 0.8	3.5 ± 1.7	0.685
5	CE nipple level, cm	Control	2.1 ± 0.6	1.2 ± 0.9	0.002
		Experimental	2.5 ± 0.9	2.0 ± 1.4	0.131
6	CE xiphisternal level, cm	Control	1.8 ± 0.5	0.6 ± 0.8	0.001
		Experimental	2.4 ± 0.9	1.5 ± 1.6	0.017

Between-group differences related to the post-intervention period are presented in Table 4. A significant difference was found between the control and experimental groups regarding post-procedure oxygen saturation (SpO₂) and respiratory rate (p = 0.001). However, there was no statistically significant difference in peak expiratory flow rate between the two groups (p = 0.85). Between-group differences in chest expansion were statistically significant only at the axillary level (p = 0.019), and there was no statistically significant improvement at the nipple (p = 0.067) and xiphisternal levels (p = 0.59).

**Table 4:** Comparison of post-intervention values of pulmonary parameters between control and experimental groups SD: standard deviation; PEFR: peak expiratory flow rate; SpO₂: oxygen saturation; RR: respiratory rate; CE: chest expansion

SI. No.	Variables	Groups	Mean ± SD	P-value
1	PEFR, L/min	Control	310 ± 59.8	0.85
		Experimental	315 ± 70.17	
2	SpO_2_, %	Control	90 ± 3.01	0.001
		Experimental	96 ± 1.94	
3	RR, breaths/min	Control	20 ± 2.01	0.001
		Experimental	18 ± 1.09	
4	CE axillary level, cm	Control	2.0 ± 1.4	0.019
		Experimental	3.5 ± 1.7	
5	CE nipple level, cm	Control	1.2 ± 0.9	0.067
		Experimental	2.0 ± 1.4	
6	CE xiphisternal level, cm	Control	0.6 ± 0.8	0.059
		Experimental	1.5 ± 1.6	

**Figure 2:**
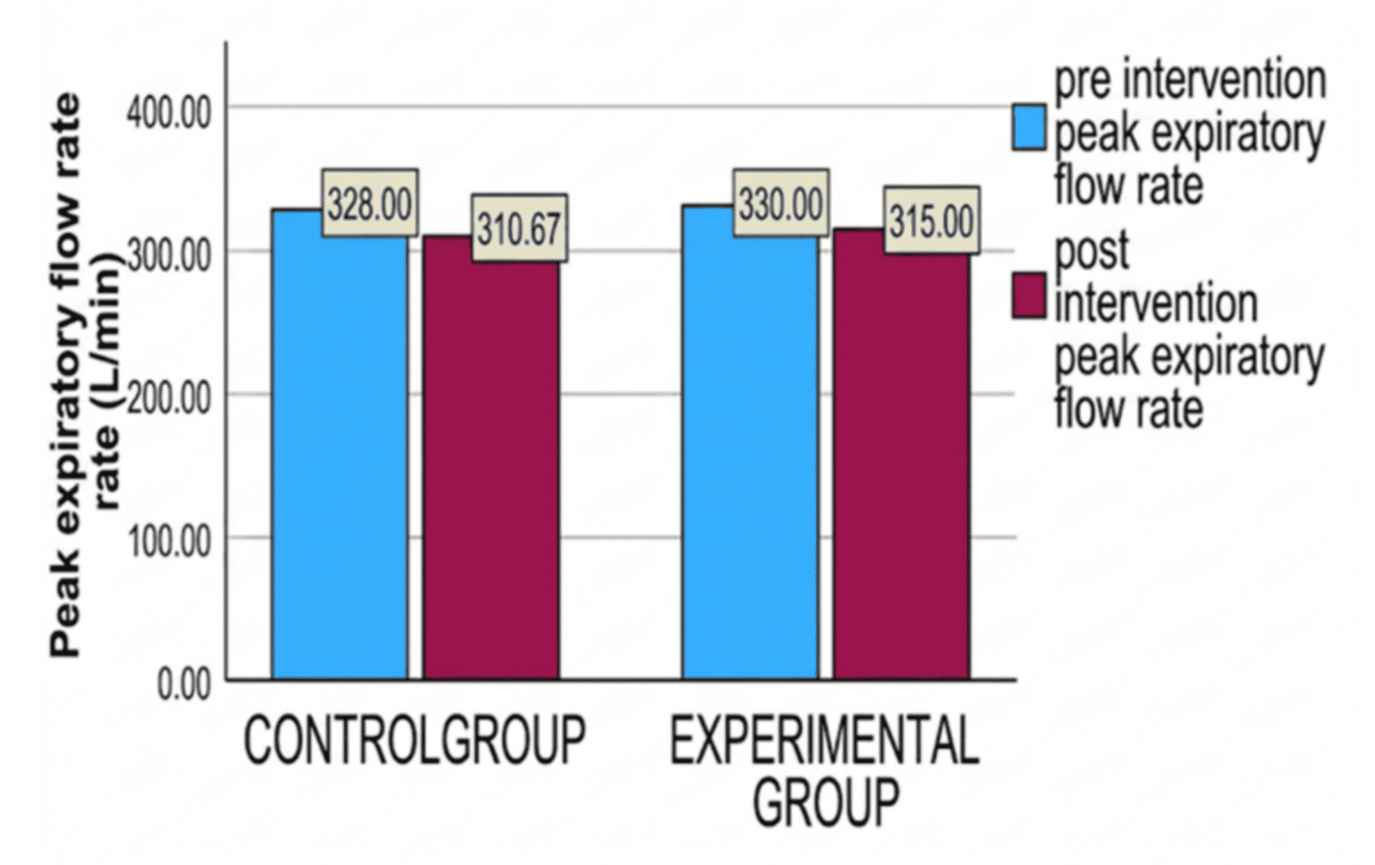
Comparison of pre-and post-intervention peak expiratory flow rate in control and experimental groups

**Figure 3:**
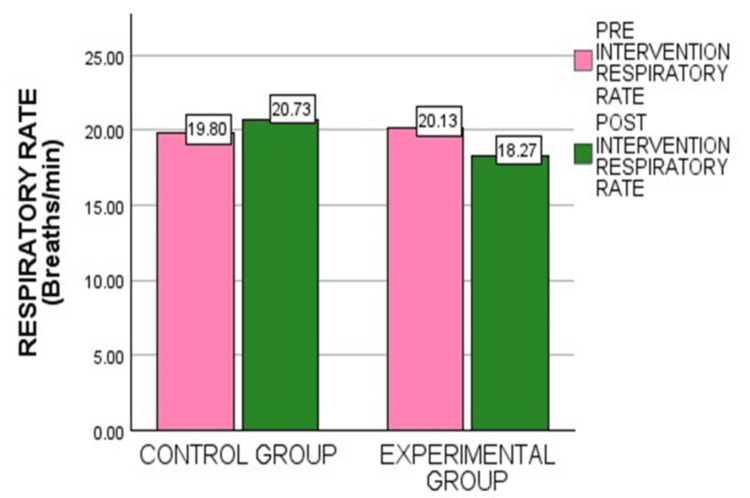
Comparison of pre-and post-intervention respiratory rate in control and experimental groups

**Figure 4:**
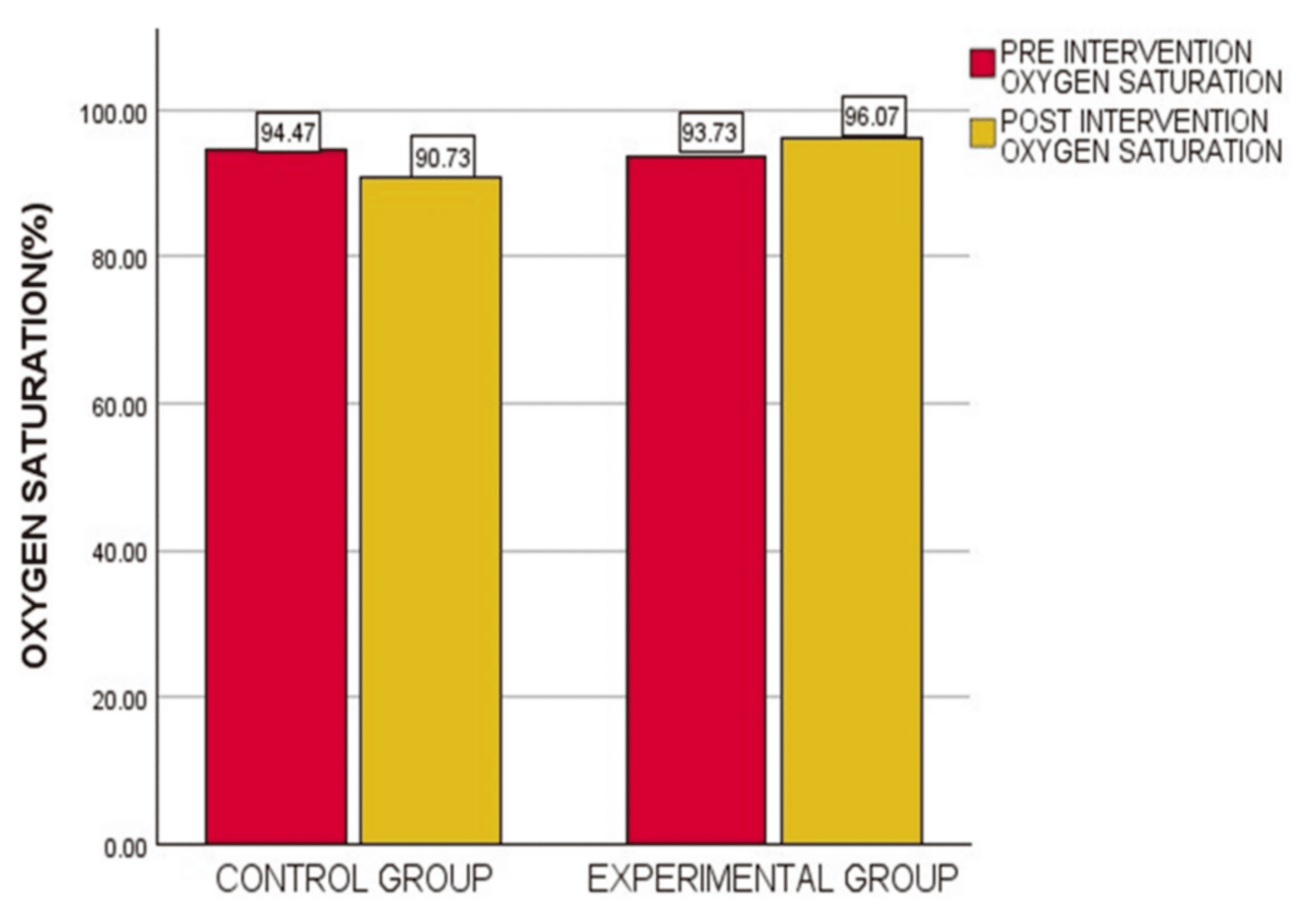
Comparison of pre-and post-intervention oxygen saturation in control and experimental groups

**Figure 5:**
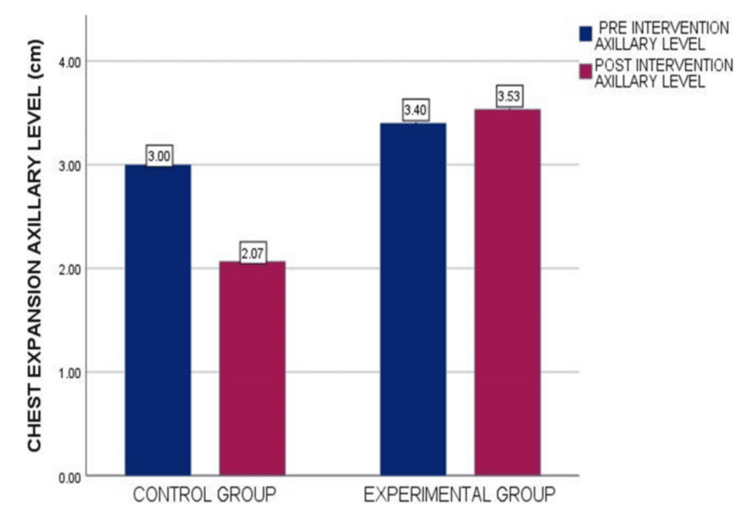
Comparison of pre-and post-intervention chest expansion at the axillary level in control and experimental groups

**Figure 6:**
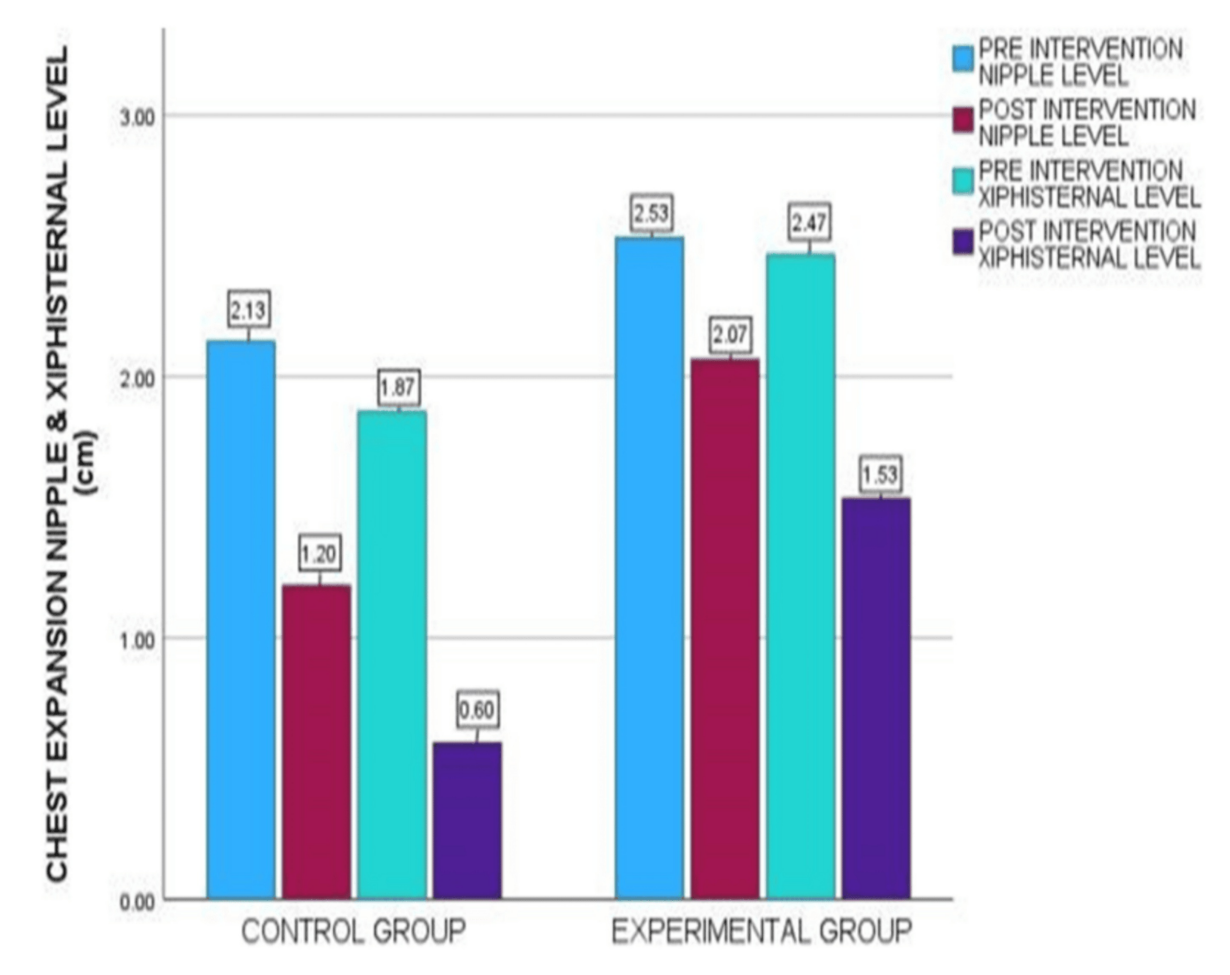
Comparison of pre-and post-intervention chest expansion nipple and xiphisternal levels in control and experimental groups

The mean values of these pre- and post-intervention outcomes are presented in the figures below. Both groups demonstrated a decrease in peak expiratory flow rate post-intervention, with no statistically significant difference between them (Figure 2). Oxygen saturation is shown in Figure 3, confirming a significant post-intervention improvement in the experimental group compared to the control group. Respiratory rate values are presented in Figure 4, showing a notable reduction in the experimental group and an increase in the control group. Regarding chest expansion at the axillary level (Figure 5), the experimental group showed greater improvement compared to the control group. At the nipple and xiphisternal levels (Figure 6), chest expansion values decreased in both the control and experimental groups post-intervention.

## Discussion

Individuals undergoing bronchoscopy commonly present with several complications, including hypoxemia, reduced oxygen saturation, arrhythmia, bronchospasm, and laryngeal spasm. This leads to an acute decline in physiological parameters of the pulmonary system. It can impair ventilation and oxygenation after the procedure. This study aimed to determine the immediate effects of pre-bronchoscopy pulmonary physiotherapy on post-procedure pulmonary function in individuals undergoing bronchoscopy.

A total of 30 individuals undergoing flexible bronchoscopy with BAL were selected and divided into two groups: the control group and the experimental group. The results demonstrate that the application of pulmonary physiotherapy techniques before bronchoscopy resulted in statistically significant improvements in selected pulmonary parameters, such as oxygen saturation and respiratory rate, in the experimental group. Peak expiratory flow rate did not show a statistically significant difference between the two groups. Chest expansion measurements at the axillary level showed significant results in both the control and experimental groups, while measurements at the other levels did not demonstrate statistically significant improvement.

Individuals in the experimental group demonstrated favorable selected pulmonary function outcomes following bronchoscopy compared to those receiving routine care in the control group. These findings may indicate that pulmonary physiotherapy exercises contributed to improved breathing patterns, increased alveolar ventilation, and a reduction in respiratory rate. The demographic characteristics and pre-intervention values of both groups appeared comparable at baseline, which minimized the potential influence of baseline differences on the study outcomes.

The relaxed diaphragmatic breathing exercise improves ventilation by promoting diaphragmatic movement. It improves breathing patterns, oxygenation, and alveolar ventilation, helps enhance chest expansion, improves gas exchange, reduces the respiratory rate, and promotes relaxation. A systematic review has shown that diaphragmatic breathing may be useful for stress management, and evidence suggests that diaphragmatic breathing enhances pulmonary function [8,13-15].

Thoracic mobility exercises, including paired trunk extension with inspiration and trunk flexion with exhalation, are simple active free exercises that can effectively increase ventilation and chest expansion. These exercises encourage the individual to breathe in; the chest wall muscles, such as the intercostal muscles, are maximally stretched, thereby improving ventilation. The improvement in oxygen saturation observed in the experimental group may be associated with enhanced alveolar ventilation, improved ventilation-perfusion matching, improved breathing efficiency, and reduced respiratory distress, which may also have contributed to the reduced respiratory rate. Low-intensity exercise, such as mobilization, can improve physiological mechanisms by increasing alveolar ventilation, tidal volume, and minute ventilation, promoting better ventilation-perfusion matching, peripheral circulation, and oxygenation, and decreasing peripheral resistance [16].

Peak expiratory flow rate did not show a statistically significant difference in the experimental group. This may be due to bronchoscopy-related irritation of the bronchial airways, which can lead to increased airway resistance, bronchial inflammation, bronchospasm, the severity of disease, and the type of respiratory condition, all of which may affect expiratory flow rate post-procedure. Short-term interventions may be more effective in improving ventilation and oxygenation rather than expiratory flow rate, which typically requires specific individualized exercises that may necessitate a longer intervention duration [17]. Chest expansion at different levels did not show significant improvement; factors such as sedation effects, underlying respiratory conditions, severity of illness, and procedure-related physiological changes may have influenced chest expansion after bronchoscopy.

There is evidence suggesting that pre-surgical procedures and rehabilitation programs may improve postoperative outcomes and reduce complications [18-19]. There are limited studies regarding pre-bronchoscopy pulmonary physiotherapy. To the best of our knowledge, this study is among the first to specifically investigate the role of pre-bronchoscopy pulmonary physiotherapy in immediate post-procedure pulmonary function outcomes. This study highlights that simple physiotherapy techniques performed before bronchoscopy are safe, cost-effective, and relatively easy to administer, making them feasible for routine clinical practice.

This study has several limitations. The sample size was relatively small, which may limit the generalizability of the findings. The quasi-experimental study design may introduce potential selection bias and affect internal validity. The intervention period was short, and only immediate post-procedure outcomes were assessed. Additionally, pulmonary function was assessed using selected pulmonary parameters; future studies may include pulmonary function test measurements for a more detailed evaluation. The findings may not be generalizable to other bronchoscopy procedures, as only individuals undergoing flexible bronchoscopy with BAL were included. Future studies with larger sample sizes, randomized controlled study designs, and longer follow-up periods are recommended to validate these findings.

## Conclusions

In this study, pre-bronchoscopy pulmonary physiotherapy demonstrated beneficial short-term effects on selected pulmonary function in individuals undergoing bronchoscopy. The findings suggest that pre-bronchoscopy pulmonary physiotherapy may support respiratory function, particularly by improving oxygen saturation and reducing respiratory rate following the bronchoscopy procedure. Techniques such as relaxed diaphragmatic breathing, thoracic mobility exercises, and low-intensity mobilization may help optimize respiratory mechanics.

## References

[1] Leiten EO, Martinsen EM, Bakke PS, Eagan TM, Grønseth R (2016). Complications and discomfort of bronchoscopy: a systematic review. Eur Clin Respir J.

[2] Batra H, Yarmus L (2018). Indications and complications of rigid bronchoscopy. Expert Rev Respir Med.

[3] Madan K, Mohan A, Agarwal R, Hadda V, Khilnani GC, Guleria R (2018). A survey of flexible bronchoscopy practices in India: the Indian Bronchoscopy Survey (2017). Lung India.

[4] Hasdiraz L, Oguzkaya F, Bilgin M, Bicer C (2006). Complications of bronchoscopy for foreign body removal: experience in 1,035 cases. Ann Saudi Med.

[5] Kim SY, Lee HJ, Lee JK (2022). Association between oxygen saturation level during bronchoscopy and post-bronchoscopy adverse events: a retrospective cohort study. Respir Res.

[6] Mohan A, Guleria R, Ali A, Kumar A (2011). Acute changes in physiological parameters and pulmonary function during and after fibreoptic bronchoscopy. Eur Respir J.

[7] Stahl DL, Richard KM, Papadimos TJ (2015). Complications of bronchoscopy: a concise synopsis. Int J Crit Illn Inj Sci.

[8] Hamasaki H (2020). Effects of diaphragmatic breathing on health: a narrative review. Medicines (Basel).

[9] Nazir S, Mathiyakom W, Tassawar MA, Tantisuwat A (2026). The effect of diaphragmatic breathing and diaphragmatic mobilization on physical performance, fear of falling, and quality of life in community-dwelling older adults: a randomized controlled trial. PLoS One.

[10] Mulero Portela AL, Colón Santaella CL, Rogers LQ, Missaghian Vissepo M (2024). Effect of low- and moderate-intensity endurance exercise on physical functioning among breast cancer survivors: a randomized controlled trial. Support Care Cancer.

[11] Priego-Jiménez S, Priego-Jiménez P, López-González M, Martinez-Rodrigo A, López-Requena A, Álvarez-Bueno C (2025). Impact of prehabilitation components on oxygen uptake of people undergoing major abdominal and cardiothoracic surgery: a network meta-analysis of randomized controlled trials. J Clin Med.

[12] Zhang G, Wang L, Han J, Chen J, Wu J (2025). Effectiveness of bronchoscopy-assisted postoperative respiratory management in patients with lung cancer and impaired cough strength: a retrospective cohort study. Ann Med Surg (Lond).

[13] Al-Reda DA, Rajha AH (2020). Effect of preoperative breathing exercise on postoperative patients’ lung functions. Indian J Forensic Med Toxicol.

[14] Prem V, Sahoo RC, Adhikari P (2013). Effect of diaphragmatic breathing exercise on quality of life in subjects with asthma: a systematic review. Physiother Theory Pract.

[15] Hopper SI, Murray SL, Ferrara LR, Singleton JK (2019). Effectiveness of diaphragmatic breathing for reducing physiological and psychological stress in adults: a quantitative systematic review. JBI Database System Rev Implement Rep.

[16] Varma VR, Tan EJ, Wang T (2014). Low-intensity walking activity is associated with better health. J Appl Gerontol.

[17] DeVrieze BW, Goldin J, Giwa AO (2024). Peak Flow Rate Measurement. https://www.ncbi.nlm.nih.gov/books/NBK459325/.

[18] Kale PM, Mohite VR, Chendake MB, Gholap MC (2017). The effectiveness of pre-operative deep breathing exercise on post-operative patients of abdominal surgery. Asian J Pharm Clin Res.

[19] Ren J, Li Z, He Y, Gao H, Li J, Tao J (2024). Systematic review and meta-analysis of breathing exercises effects on lung function and quality of life in postoperative lung cancer patients. J Thorac Dis.

